# Supplementary material for: Robust recognition and exploratory analysis of crystal structures via Bayesian deep learning
Source: Nat Commun. 2021 Oct 29;12:6234. doi: 10.1038/s41467-021-26511-5 (PMC8556392; doi:10.1038/s41467-021-26511-5)
Supplement: Supplementary file 1 — Supplementary Information [file 41467_2021_26511_MOESM1_ESM.pdf]

Robust recognition and exploratory analysis of crystal structures via Bayesian deep learning

Andreas Leitherer et al.

Supplementary Methods

Isotropic scaling. To reduce the dependency on lattice parameters, we isotropically scale each prototype according to its nearest neighbor distance d_{NN}. This way, one degree of freedom is eliminated, implying that all cubic systems are equivalent and thus are correctly classified by construction. To compute d_{NN}, we calculate in a first step the histogram of all nearest neighbor distances. Since the area of spherical shells grows with the squared radius, we divide the histogram by the squared radial distance. Then, we use the center of the maximally populated bin as the nearest neighbor distance d_{NN}. Dividing the atomic position by d_{NN} yields the final isotropically scaled structure, which is used for calculating the SOAP descriptor. Alternatively, one may use the mean of the nearest neighbors as d_{NN}, which, however, is more prone to defects. In case of multiple chemical species, we consider all possible substructures as formed by the constituting species to calculate the SOAP descriptor (see next paragraph). For each of the substructures, we compute d_{NN}, while we determine the histogram of neighbor distances only from distances between atoms whose chemical species coincide with those of the substructure. For instance, given the substructure (α, β), i.e., the atomic arrangement of atoms with species β as seen from the perspective of atoms with species α, we consider only α-atoms and determine all distances to β-atoms.

SOAP descriptor. As discussed in the main text, encoding of physical requirements we know to be true is crucial for machine-learning application. For instance, in crystal classification, two atomic structures that differ only by a rotation must have the same classification label. This is not guaranteed if real space atomic coordinates are used as descriptor (cf. Fig. 1a). As an attempt to fix this, one might include a discrete subset of orientations in the training set, hoping that the model will generalize to unseen rotations. However, there is no theoretically guarantee that the model will learn the rotational symmetry, and if it does not, it will fail to generalize and return different predictions for symmetrically equivalent structures. In contrast, when a rotationally invariant descriptor is employed, only one crystal orientation needs to be included in the training set and the model will generalize to all rotations by construction. This reasoning readily applies to other physics requirements such as translational, or permutation invariance (for atoms with same chemical species).

In the following, we provide details on adapting the standard SOAP descriptor such that its number of components is independent on the number of atoms and chemical species.

Starting with the simple case of one chemical species, we consider a local atomic environment $\mathscr{X}$, being defined by a cutoff region (with radius R_{C}) around a central atom, located at the origin of the reference frame. Each atom within this area is represented by a Gaussian function centered at the atomic position $\mathbf{r}_i$ and with width σ. Then, the local atomic density function of $\mathscr{X}$ can be written as[1]

$$\rho_{\mathscr{X}}(\mathbf{r}) = \sum_{i\in\mathscr{X}} \exp\left(-\frac{(\mathbf{r}-\mathbf{r}_i)^2}{2\sigma^2}\right) = \sum_{blm} c_{blm} u_b(r) Y_{lm}(\hat{\mathbf{r}}), \quad (1)$$

where in the second step, an expansion in terms of spherical harmonics $Y_{lm}(\hat{\mathbf{r}})$ and a set of radial basis functions $\{u_b(r)\}$ is performed. One can show that the rotationally invariant power spectrum is given by[1]

$$p(\mathscr{X})_{b_1 b_2 l} = \pi\sqrt{\frac{8}{2l+1}}\sum_m (c_{b_1 lm})^{\dagger} c_{b_2 lm}. \quad (2)$$

These coefficients can be arranged in a normalized (SOAP) vector $\hat{\mathbf{p}}\,(\mathscr{X})$, describing the local atomic environment $\mathscr{X}$. In total, we obtain as many SOAP vectors as atoms in the structure, which one can average to obtain a materials descriptor independent of the number atoms N_{at}. Another possibility (the standard setting in the software we use) is to average the coefficients c_{blm} first and then compute Eq. 2 from this[2]. The cutoff radius R_{C} and σ (cf. Eq. 1) are hyperparameters, i.e., supervised learning cannot be used directly to assign values to these parameters, while their specific choice will affect the results. Typically, one would employ cross-validation while here, we take a different route: First, we assess the similarity between SOAP descriptors using the cosine similarity to identify parameter ranges that provide sufficient contrast between the prototypes. Using this experimental approach, we find that values near $\sigma = 0.1 \cdot d_{\mathrm{NN}}$ and $R_{\mathrm{C}} = 4.0 \cdot d_{\mathrm{NN}}$ yield good results. Then we augment our dataset with SOAP descriptors calculated for different parameter settings.

The extension to several chemical species is achieved by considering all possible substructures as formed by the constituting atoms: Considering NaCl, we first inspect the lattice of Cl atoms as seen by the Na atoms, which we denote by $(\mathrm{Na},\mathrm{Cl})$; this means that Na atoms are considered as central atoms in the construction of the local atomic environment while only Cl atoms are considered as neighbors. A similar construction is made for the remaining substructures $(\mathrm{Na},\mathrm{Na})$, $(\mathrm{Cl},\mathrm{Na})$, and $(\mathrm{Cl},\mathrm{Cl})$, which may be quite similar depending on the atomic structure. For each substructure, we compute the SOAP vectors via Eq. 2, obtaining a collection of SOAP vectors. Averaging these gives us four (in case of NaCl) averaged SOAP vectors. Averaging the latter again, yields a materials representation being independent on the number of atoms and chemical species.

Formally, given a structure with S species $\alpha_1, ..., \alpha_S$, we consider all substructures formed by pairs of species $(\alpha_i, \alpha_j), j = 1, ..., S$, resulting in S^2 averaged SOAP vectors $< \hat{\mathbf{p}}_{\alpha_i\alpha_j} >_{N_{\mathrm{at},\alpha_i}}$, where the bracket represents the average over number of atoms N_{at} of species α_i. These vectors are averaged over, yielding the final vectorial descriptor $<< \hat{\mathbf{p}}_{\alpha_i\alpha_j} >>_{\alpha_i\alpha_j}$.

Note that this construction of SOAP deviates from the previously reported way of treating multiple chemical species in the following way: Usually, for each atom, one constructs the

following power spectra[3]

$$p(\mathscr{X})^{\alpha\beta}_{b_1 b_2 l} = \pi\sqrt{\frac{8}{2l+1}}\sum_m (c^{\alpha}_{b_1 lm})^{\dagger} c^{\beta}_{b_2 lm}, \tag{3}$$

where the coefficients originate from basis set expansion as in Eq. 2, while the density ρ is constructed separately for each species. For a specific α and β, the coefficients of Eq. 3 can be collected into vectors $\mathbf{p}_{\alpha\beta}$. In case of $\alpha \neq \beta$, cross-correlations, i.e., products of coefficients from different densities are used to construct the vectors $\mathbf{p}_{\alpha\beta}$, which are missing in our version.

Bayesian deep learning. As discussed in the main text, one can think of Bayesian neural networks as standard neural networks with distributions being placed placed over the model parameters. This results in probabilistic outputs from which principled uncertainty estimates can be obtained. The major drawback is that training and obtaining predictions from traditional Bayesian neural networks is generally difficult because it requires solving computationally costly high-dimensional integrals. For classification, expensive calculations are required to determine $p(y = c|x, \mathrm{D}_{\mathrm{train}})$, which is the probability that the classification is assigned to a class c, given input x and training data $\mathrm{D}_{\mathrm{train}}$. Then, for a specific input x (in our case the SOAP descriptor), the most likely class c, i.e., the one with largest $p(y = c|x, \mathrm{D}_{\mathrm{train}})$ is the predicted class.

Gal and Ghahramani[4] showed that stochastic regularization techniques such as dropout[5,6] can be used to calculate high-quality uncertainty estimates (alongside predictions) at low cost. In dropout, neurons are randomly dropped in each layer before the network is evaluated for a given input. Usually, dropout is only used at training time with the goal of avoiding overfitting by preventing over-specialization of individual units. Keeping regularization also at test time allows to quantify the uncertainty. Practically, given a new input, one collects and subsequently aggregates the predictions while using dropout at prediction time. This gives a collection of probabilities being denoted as $p(y = c|x, \omega_t)$, which is the probability of predicting class c given the input x at a specific forward-pass t, with model parameters ω_t. From this collections of probabilities, one can estimate the actual quantity of interest, $p(y = c|x, \mathrm{D}_{\mathrm{train}})$, by a simple average[4]:

$$p(y = c|x, \mathrm{D}_{\mathrm{train}}) \approx \frac{1}{T}\sum_{t=1}^{T} p(y = c|x, \omega_t), \tag{4}$$

where T is the number of forward-passes (see Methods section "Neural network architecture and training procedure" for details on how we choose this parameter). While the average can be used to infer the class label c, additional statistical information, which reflects the predictive uncertainty, is contained in the collected forward-passes, i.e., the probabilities $p(y = c|x, \omega_t)$ which effectively yield a histogram for each class and define, when varying over all possible c, a (discrete) probability distribution. For instance, mutual information can be used to quantify the uncertainty from the expressions $p(y = c|x, \omega_t)$. Specifically, for a given test point x, the mutual information between the predictions and the model posterior $p(\omega|\mathrm{D}_{\mathrm{train}})$ (which captures the most probable parameters given the training data) is defined as[4,7]

$$\begin{aligned}&\mathbb{I}[y, \omega|x, \mathrm{D}_{\mathrm{train}}] \approx \\ &-\sum_c \left(\frac{1}{T}\sum_t p(y = c|x, \omega_t)\right)\log\left(\frac{1}{T}\sum_t p(y = c|x, \omega_t)\right) \\ &+\frac{1}{T}\sum_c\sum_t p(y = c|x, \omega_t)\log p(y = c|x, \omega_t).\end{aligned} \tag{5}$$

Hyperparameter optimization. The Tree-structured Parzen estimator (TPE) algorithm[8,9] is an example of a Bayesian optimization technique. Specifically, one has to define a search space which can comprise a variety of parameters such as the learning rate or model size specifics such as the number of layers or neurons. Then, the goal is to minimize a performance metric (in our case, we maximize the accuracy by minimizing its negative). For large search spaces, iterating through each possible combination, i.e., performing a grid search, will get expensive very quickly. Random search is one alternative, while Bayesian methods such as TPE can be more efficient. Approaches such as grid or random search assign uniform probability to each hyperparameter choice, which implies that a long time is spent at settings with low reward. This becomes particularly troublesome if the performance metric is expensive to calculate. In Bayesian methods such as TPE, the objective is replaced by a computationally cheaper surrogate model (for instance, Gaussian process or random forest regressor). New hyperparameters are selected iteratively in a Bayesian fashion. Specifically, the selection is based on an evaluation function (typically so-called expected improvement) taking into account the history of hyperparameter selections and thus avoiding corners of the search space with low reward.

The search space is chosen the following way (alongside the chosen hyperopt commands hp.choice or hp.uniform):

- Number of layers (2, 3, 4, 5), hp.choice
- Number of neurons in each layer (256 or 512), hp.choice
- Batch size, (64 or 128), hp.choice
- Learning rate, range (0.01,0.0001), hp.uniform
- Dropout rate, range (0.01, 0.05), hp.uniform

Supplementary Notes

Supplementary Note 1

In the following, we provide details on the benchmarking.

For spglib, we only include prototypes from AFLOW. The reason for excluding structures from the computational materials repository (CMR) is that we do not always have the correct or meaningful labels for all structures. For instance, some 2D materials are specified as P1 in the database, which

cannot be used as a correct label. Furthermore, for quaternary chalcogenides, the expected symmetries (as specified in the corresponding reference[10]) cannot be reconstructed, which is most likely due to local optimization effects. Similar observations were made for the ternary Perovskites. More careful choice of precision parameters or additional local optimization may help. Thus, to enable a fair comparison, the benchmarking in the main text only reports results on elemental and binary compounds from AFLOW (where we know the true labels), while the performance on both AFLOW and CMR data is shown in Supplementary Tables 1, 2, and 3. To avoid the impression that spglib is not applicable to ternary, quaternary, and 2D materials, we still provide the label "96/108" behind spglib methods in the benchmarking tables. Note that non-periodic structures are excluded for benchmarking (again only in the main table), in particular carbon nanotubes, since these systems cannot be treated by spglib.

For the other benchmarking methods, which are common neighbor analysis (CNA, a-CNA), bond angle analysis (BAA), and polyhedral template matching (PTM), we use implementations provided in OVITO[11], where for BAA we apply the Ackland jones method. As for spglib, only periodic structures were included. BAA, CNA, a-CNA all include fcc, bcc, and hcp structures, while PTM contains in addition sc, diamond, hexagonal diamond, graphene, graphitic boron nitride, L10, L12, zinc blende, and wurtzite. Each of the frameworks provide one label for each atom, i.e., for a structure with N atoms we obtain N labels. To obtain an accuracy score, we compare these N predictions to N true labels, which correspond to the space group associated with the prototype label (e.g., 194 for hcp). For CNA, we select the standard cutoff (depending on its value one is able to detect bcc but not hcp and vice versa). Also for BAA (Ackland jones) and a-CNA standard settings are used. For PTM, an RMSD cutoff of 0.1 was used (again default in OVITO). Note that PTM can also distinguish different sites of the L12 structure. For simplicity, we did not label the L12 structure by sites and take this classification into account, but always assign a true label as soon as an atom was assigned to the L12 class (even if it might be not the correct site).

Furthermore, for ARISE periodic and non-periodic structures are included, while for the benchmarking methods only periodic structures are considered. While for spglib, translational symmetry is violated by construction, the other methods can in principle be applied to these systems. However, when calculating the accuracy for a given non-periodic structure, we have to choose a label for the boundary atoms. If we select the same label for these atoms as for the central ones (which have a sufficiently larger number of neighbors), these methods will usually predict the class "None" and interpreting this as a "misclassification" would decrease the total classification accuracy. Therefore, for a fair comparison, we exclude non-periodic structures.

Supplementary Note 2

The Bain transformation path describes a structural transitions between bcc and fcc symmetries via intermediate tetragonal phases[12] of body-centered – or equivalently – face-centered tetragonal symmetry. Originally investigated for iron[12], the Bain path is relevant in thermo-mechanical processing – a central aspect for steel properties[13] – as it serves as a model for temperature-induced transitions between fcc (γ) and bcc (α) iron[14]. The Bain path is also crucial for understanding properties of epitaxial films[15,16] or metal nanowires[17].

Practically, the structures constituting a Bain path can be obtained by varying the ratio c/a between lattice parameters a and c of a tetragonal structure (cf. Supplementary Fig. 1a); $c/a = 1$ corresponds to a cubic structure. We generate tetragonal geometries for lattice parameters a, c taking values in $[3.0\,\text{Å}, 6.0\,\text{Å}]$ with steps of 0.05 Å, resulting in 3721 crystal structures. These structures are then classified with ARISE, and the results depicted via classification and uncertainty maps in Supplementary Fig. 1b and c, respectively. Each point in these maps corresponds to a prediction for a specific geometry. We include in the training set fcc, bcc, and tetragonal geometries with structural parameters known experimentally; they are shown as stars in Supplementary Fig. 1b. Specifically, the lattice parameters $(a, c, c/a)$ are $(3.155\text{Å}, 3.155\text{Å}, 1.0)$ for the bcc[18] and $(3.615\text{Å}, 5.112\text{Å}, \sqrt{2})$ for the fcc prototype[19], while two tetragonal structures (being assigned one common label "tetragonal") are included with $(3.253\text{Å}, 4.946, \text{Å}, 1.521)$ in case of In[20] and $(3.932\text{Å}, 3.238\text{Å}, 0.824)$ for $\alpha - \text{Pa}$[21]. We isotropically scale every geometry to remove one degree of freedom (see Supplementary Methods section), so that all possible cubic lattices are effectively equivalent; this allows the model to generalize by construction to all cubic lattices regardless of the lattice parameter. The same holds for tetragonal structures (i.e., two degrees of freedom) with constant c/a ratio. As visual aid, we mark lines of constant c/a in Supplementary Fig. 1b-c starting from the four structures included in the training set. Note that any path connecting the constant c/a ratios corresponding to fcc and bcc structures constitutes a Bain path. To obtain a classification label, we select the class with the higher classification probability through a so-called argmax operation (i.e., the label c maximizing Eq. 4). These predictions are shown in Supplementary Fig. 1.

The model is able to detect the bcc and fcc phases in the expected areas, while all prototypes not being fcc, bcc, or tetragonal are correctly labeled as "Other". We point out that only four structures – corresponding to points in the plot marked by the four stars – are included in the training set, while all other 3717 structures are model (test) predictions. We can also observe that the model correctly predict the presence of a a tetragonal phase between fcc (yellow band) and bcc (green band), even though no tetragonal structures from this region are included in the training set. This transition is smooth, only interrupted by small areas for which other, low-symmetry prototypes are assigned, but with high uncertainty, as quantified by the mutual information, cf. Supplementary Fig. 1 c. We provide the classification probabilities of all assigned prototypes in Supplementary Fig. 3. In general, increased uncertainty appears at transitions between the assignments of different prototypes. We also note that there is a smooth transition for classification probabilities at the transition between prototypes (cf. Supplementary Fig. 3). These results represent a first indication that the network has learned physically meaningful representations. Surprisingly, for large or small c/a ratios, i.e., points far outside the training set, other (low-symmetry) phases appear such as base-centered orthorhombic molecular iodine or face-centered orthorhombic $\gamma-$Pu with small uncertainty. While it may be desirable to avoid overconfident predictions far away from the training set, the assignments could be actually physically justified given the similarities between tetragonal and orthorhombic lattices, the most evident being that all angles in both crystal systems are $90°$. We note that the transition to these prototypes is encompassed by regions of high uncertainty also in this case in agreement with physical intuition.

Supplementary Note 3

In the following, we investigate scenarios in which the model is forced to fail, i.e., we analyze ARISE out-of-sample predictions.

To assess the physical content learned by the network, we investigate its predictions – and thus its generalization ability – on structures corresponding to prototypes not included in the training. This is of particular relevance if one wants to use predictions of ARISE – for applications such as screening of large databases, or create low-dimensional maps for a vast collection of materials[22].

Given an unknown structure, the network needs to decide – among the classes it has been trained on – which one is the most suitable. It will assign the most similar prototypes and quantify the similarity via classification probabilities, providing a ranking of candidate prototypes. The uncertainty in the assignment, as quantified by mutual information, measures the reliability of the prediction. Note that the task of assigning the most similar prototype(s) to a given structure among 108 possible classes (and quantifying the similarity) is a very complicated task even for trained materials scientists, in particular in case of complex periodic and possibly defective three-dimensional structures.

We consider three examples (cf. Supplementary Fig. 2 left): fluorite and tungsten Carbide (from AFLOW) where the correct labels are known, and one structure from the NOMAD encyclopedia (see last paragraph of this section for details on the provenance), for which the assigned space group is 1, i.e., no symmetry can be identified (via spglib). In all three cases there is no prototype in the dataset which represent a match for any of these structures. This is on purpose: the network will "fail" by construction since the correct class is not included in the possible classes the network knows (and needs to choose from). Analyzing how the network fails will

	Pristine	Random displacements (δ)					Missing atoms (η)			
		0.1%	0.6%	1%	2%	4%	1%	5%	10%	20%
Spglib (loose)	100.00	100.00	100.00	95.26	0.20	0.00	11.23	0.00	0.00	0.00
Spglib* (loose)	67.71	67.71	67.71	65.83	14.51	0.00	15.73	0.03	0.00	0.00
Spglib (tight)	100.00	0.00	0.00	0.00	0.00	0.00	11.23	0.00	0.00	0.00
Spglib* (tight)	83.33	0.00	0.00	0.00	0.00	0.00	17.53	0.00	0.00	0.00
PTM	100.00	100.00	100.00	100.00	100.00	100.00	88.67	51.76	25.93	6.24
PTM*	8.78	11.37	11.37	11.37	11.37	11.37	10.08	5.90	2.96	0.71
CNA	66.14	62.81	62.81	54.55	32.34	31.41	55.86	32.50	15.75	3.07
CNA*	1.44	1.62	1.62	1.40	0.83	0.81	1.44	0.84	0.41	0.08
a-CNA	100.0	100.0	100.0	100.0	100.0	100.0	89.25	52.81	25.92	5.37
a-CNA*	2.49	3.08	3.08	3.08	3.08	3.08	2.75	1.64	0.81	0.17
BAA	100.0	100.0	100.0	100.0	100.0	97.85	99.71	88.78	65.21	25.38
BAA*	2.49	3.08	3.08	3.08	3.08	3.03	3.08	2.74	2.02	0.81
GNB	62.63	56.50	55.94	55.56	54.98	52.72	54.51	52.94	52.67	52.09
BNB	75.76	65.56	65.19	63.61	61.58	56.58	65.49	64.00	62.43	60.48
**ARISE** (this work)	100.00	100.00	100.00	100.00	99.86	99.29	100.00	100.00	100.00	99.85

Supplementary Table 1. Accuracy in identifying the parent class of defective crystal structures. Two lines are shown for each of the methods used for benchmarking (spglib, PTM, CNA, a-CNA, BAA): In rows without stars, the accuracy is calculated only for structures for which the respective method was designed for; for instance, spglib can be applied to every structure of Fig. 1e except the 12 nanotubes (note that we only include prototypes from AFLOW for spglib, cf. Supplementary Note 1). This is also true for the other methods, while additional structures have to be removed for instance for CNA, a-CNA, and BAA as they cannot classify simple cubic and diamond structures. In starred rows, all 108 classes summarized in Fig. 1e are included, leading to the strong decrease in performance. In contrast, the neural network approach proposed here can be applied to all classes, and thus the whole dataset was used. Moreover, we compare ARISE to a standard Bayesian approach: Naive Bayes (NB). We consider two different variants of NB: Bernoulli NB (BNB) and Gaussian NB (GNB), where the whole dataset was used – see the Methods section for more details. ARISE is overwhelmingly more accurate than both NB methods, for both pristine and defective structures.

	Random displacements (δ)		Missing atoms (η)	
	7%	10%	25%	30%
Spglib (loose)	0.00	0.00	0.00	0.00
Spglib* (loose)	0.00	0.00	0.00	0.00
Spglib (tight)	0.00	0.00	0.00	0.00
Spglib* (tight)	0.00	0.00	0.00	0.00
PTM	100.00	94.34	3.33	1.72
PTM*	11.37	10.71	0.38	0.19
CNA	31.41	24.20	1.38	0.55
CNA*	0.81	0.62	0.04	0.01
a-CNA	99.99	94.55	2.60	1.03
a-CNA*	3.08	2.90	0.08	0.03
BAA	87.79	69.68	14.25	7.35
BAA*	2.77	2.22	0.49	0.30
GNB	50.73	48.62	51.33	50.32
BNB	48.81	43.28	59.78	58.18
**ARISE** (this work)	97.82	94.56	99.86	99.76

Supplementary Table 2. Accuracy in identifying the parent class of defective crystal structures for high displacements (percentage δ) and missing atoms (percentage η).

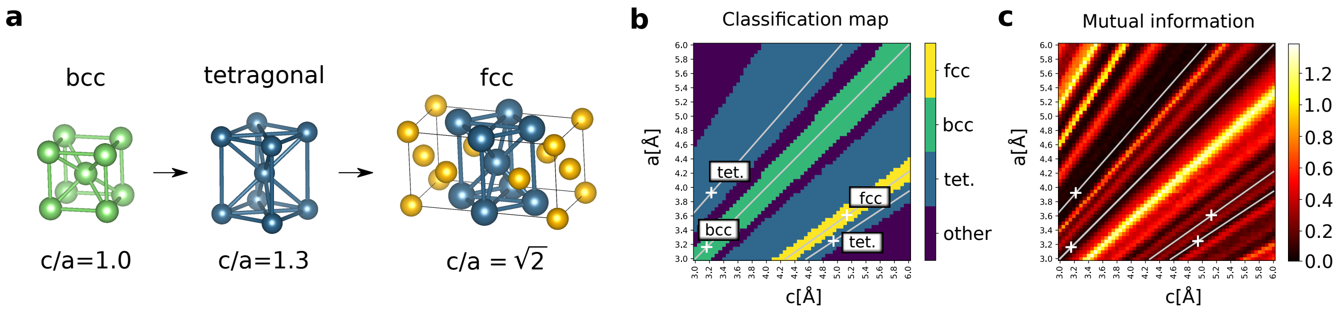

Supplementary Figure 1. Application of ARISE for the characterization of the Bain path. **a** Structures occurring in the Bain path, obtained by varying c/a; increasing c/a from 1.0 (bcc) leads to transitions to tetragonal phases and finally to the fcc structure (c/a=$\sqrt{2}$) . **b** Classification (argmax predictions, left) and uncertainty (mutual information, right) for geometries in the range $c, a \in [3.0\,\text{Å}, 6.0\,\text{Å}]$. Geometries included in the training set are marked by stars in **b,c**. As we isotropically scale the structures, geometries with constant c/a are equivalent, which is indicated by solid lines.

	Missing atoms and displacements (η, δ)						
	(1%, 0.1%)	(5%, 0.6%)	(10%, 1%)	(15%,2%)	(20%,4%)	(25%,7%)	(30%,10%)
Spglib (loose)	11.32	0.00	0.00	0.00	0.00	0.00	0.00
Spglib* (loose)	15.76	0.00	0.00	0.00	0.00	0.00	0.00
Spglib (tight)	0.00	0.00	0.00	0.00	0.00	0.00	0.00
Spglib* (tight)	0.00	0.00	0.00	0.00	0.00	0.00	0.00
PTM	88.68	51.78	25.60	12.75	6.41	3.19	1.46
PTM*	10.08	5.90	2.92	1.45	0.73	0.36	0.16
CNA	55.77	31.95	13.83	4.41	2.03	0.79	0.19
CNA*	1.44	0.82	0.36	0.11	0.05	0.02	0.00
a-CNA	89.21	52.36	26.01	12.13	6.07	2.40	0.97
a-CNA*	2.75	1.62	0.81	0.38	0.19	0.08	0.03
BAA	99.72	88.98	65.17	42.62	25.95	15.58	6.63
BAA*	3.07	2.75	2.02	1.34	0.82	0.50	0.22
GNB	54.92	52.86	52.11	50.70	49.92	47.94	42.65
BNB	64.44	61.79	58.86	56.26	52.31	45.03	40.14
**ARISE** (this work)	100.00	100.00	100.00	99.88	99.29	97.31	92.50

Supplementary Table 3. Accuracy in identifying the parent class of defective crystal structures, with both missing atoms (percentage η) and displacements (percentage δ) introduced at the same time. The results show that ARISE is also robust for highly defective structures where displacements and missing atoms are present at the same time.

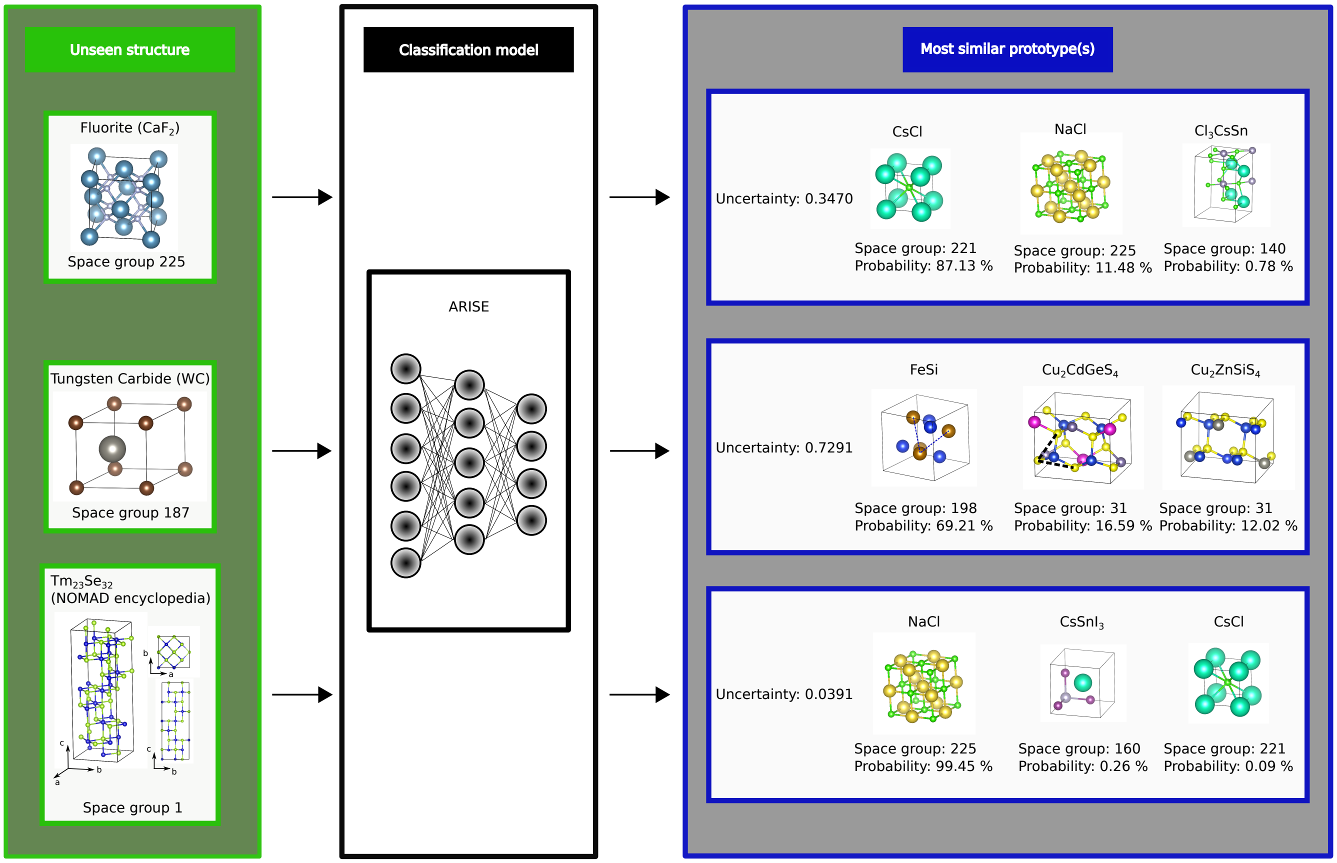

Supplementary Figure 2. Three examples for assigning the most similar prototype(s) (right panel) to structures for which the corresponding structural class is not contained in the training set of ARISE (left panel). For each prediction, space group and classification probabilities of the top predictions are specified together with an uncertainty estimate (mutual information). The space groups are returned via spglib, where we choose the highest symmetry that is found for all combinations of precision parameters $(0.1, 0.01, 0.001, 0.0001)$[Å] and angle tolerances $(1, 2, 3, 4, 5)$[°].

give us insight on the physical content of the learned model. This test can also be viewed as discovering “unexpected similarities” across materials of different chemical composition and dimensionality.

Following the pipeline for single-crystal classification summarized in Fig. 1, we compute classification probabilities and mutual information, yielding the assignments shown in Supplementary Fig. 2 right. To rationalize the predictions shown in Supplementary Fig. 2 from a physical standpoint, we inspect the substructures formed by the chemical species in both original and assigned structures. This is motivated by our choice of materials representation as averaged SOAP descriptor of substructures (see Supplementary Methods for more details). The two most similar prototypes to fluorite (CaF_2) are CsCl and NaCl, both consisting of two inter-penetrating lattices of the same type, two sc lattices for CsCl and two fcc lattices for NaCl. Fluorite contains both sc (F atoms) and fcc (Ca atoms) which is likely why CsCl and NaCl are assigned, together with a ternary halide tetragonal perovskite, also containing sc symmetry (via Cs and Sn atoms, respectively).

For tungsten carbide (WC), W and C form two hexagonal lattices. In the unit cell of the most similar prototype, FeSi, 60° angles are formed within the substructures of each species (see dashed lines in the unit cell), thus justifying this classification. Furthermore, two quaternary chalcogenides appear as further candidates. This similarity – hard to assess by eye – originates by the presence of angles close to 60° for *S* atoms (yellow) for both Cu_2CdGeS_4 and Cu_2ZnSiS_4 (marked in the figure for Cu_2CdGeS_4). Also note that these two quaternary prototypes, Cu_2ZnSiS_4 and Cu_2CdGeS_4 are a result of substituting Ge and Si with isoelectric elements Zn and Cd, which implies that these structures are expected to be similar. This explains why they both appear as candidates for structures being similar to tungsten carbide. Finally, for the compound $Tm_{23}Se_{32}$ from the NOMAD encyclopedia, the model identifies NaCl as the most similar prototype. Looking at the structure from different angles, especially from the top (cf. Supplementary Fig. 2, left part), a similarity to cubic systems can be identified. The classification method robustness to missing atoms makes the apparent gaps in the side-view negligible, and thus rationalizes the NaCl assignment. Regarding the uncertainty quantification (via mutual information), in-

creased uncertainties appear for fluorite and tungsten carbide, since besides the top prediction with more than 70% classification probability, other prototypes are possible candidates for the most similar prototype. For the NOMAD structure $Tm_{23}Se_{32}$, the network is quite confident, most likely because no other good candidates are presented among the binaries included in the 108 classes dataset.

These results show that the model – even when forced to fail by construction – returns (highly non-trivial) physically meaningful predictions. This makes ARISE particularly attractive for screening large and structurally diverse databases, in particular assessing structures for which no symmetry label can be obtained with any of the current state-of-the-art methods.

In addition to the analysis in Supplementary Fig. 2, we present some results for further out-of-sample structures:

- Boron nitride (bulk, graphitic, http://aflowlib.org/CrystalDatabase/AB_hP4_194_c_d.html) classified as hexagonal graphite (probability 63.32%), mut.inf. 0.7278

- Cementite (http://aflowlib.org/CrystalDatabase/AB3_oP16_62_c_cd.html) classified as MnP with probability 49.14%, mut.inf. 0.7176

- $CuTi_3$ (L60 Srukturbericht, http://aflowlib.org/CrystalDatabase/AB3_tP4_123_a_ce.html) classified as bct α-Pa with probability 78.41%, mut.inf. 0.8539

- Benzene (http://aflowlib.org/CrystalDatabase/AB_oP48_61_3c_3c.html) classified as nanotube (chiral indices (n,m)=(5,2)) with probability 68.48%, mut.inf. 0.6249

- Rutile (http://aflowlib.org/CrystalDatabase/A2B_tP6_136_f_a.html) classified as orthorhombic halide perovskite with probability 44.62%, mut.inf. 0.8733

- NbO (http://aflowlib.org/CrystalDatabase/AB_cP6_221_c_d.html), which is NaCl with 25% ordered vacancies on both the Na and Cl sites, classified as NaCl with probability 99.96%, mut.inf. 0.0027

- Moissanite 4H SiC (http://aflowlib.org/CrystalDatabase/AB_hP8_186_ab_ab.html) classified as wurtzite with probability 99.74%, mut.inf. 0.0166

- K_2PtCl_6 (http://aflowlib.org/CrystalDatabase/A6B2C_cF36_225_e_c_a.html) classified as NaCl with probability 61.4%, mut.inf. 0.5402

The structure taken from the NOMAD encyclopedia has the ID mp-684691 in Materials project, where further details can be found, e.g., on on the experimental origin.

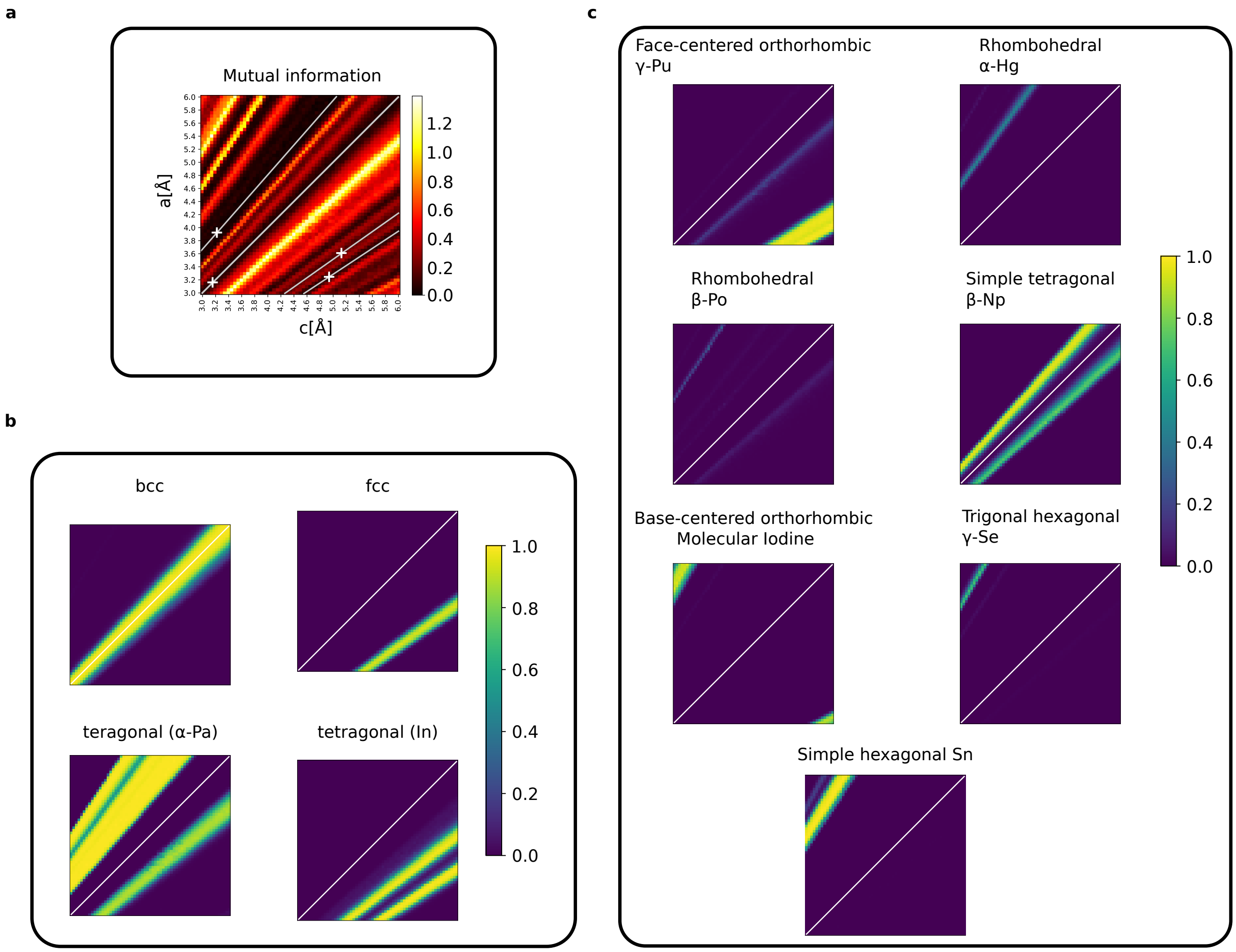

Supplementary Figure 3. Bain path - all prototypes with increased classification probability: **a** Mutual information plot showing the spots of high and low uncertainty for different geometries. **b** Classification probability maps corresponding to bcc, fcc and two tetragonal phases. **c** Representative selection of other prototypes showing non-zero classification probabilities.

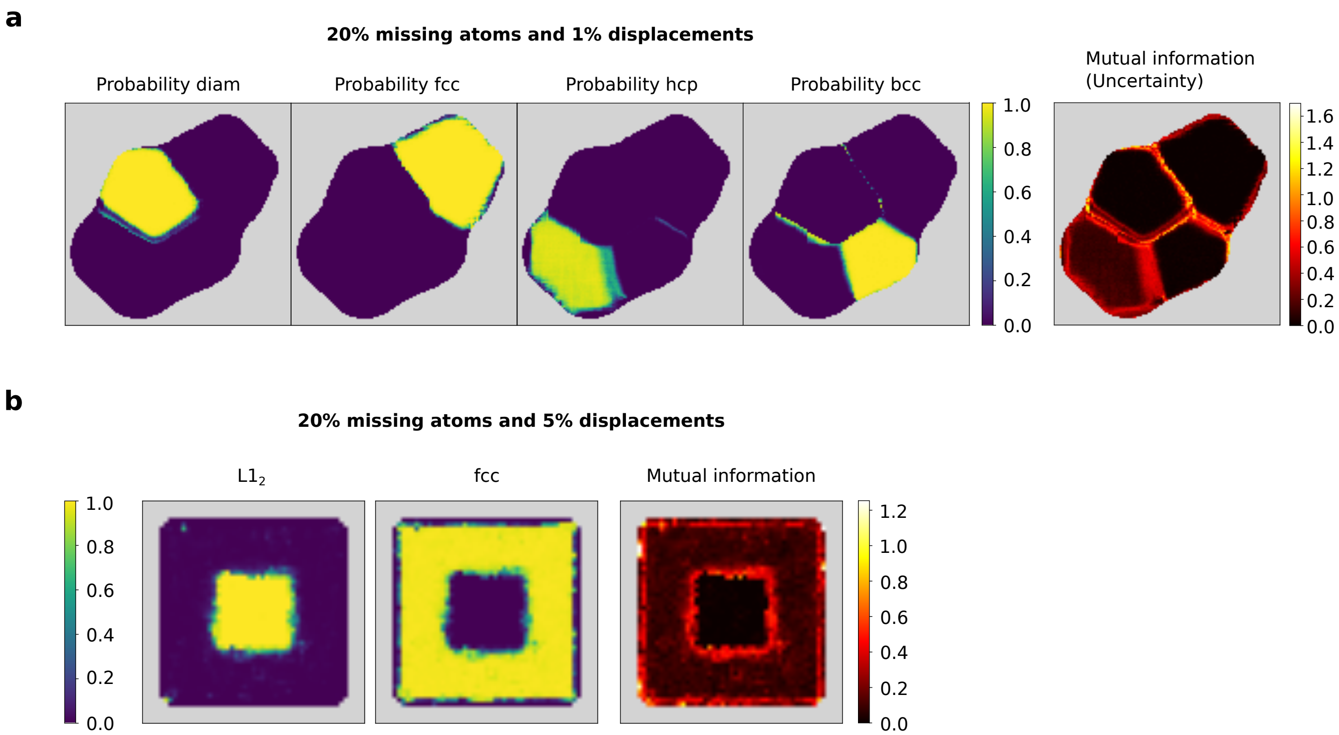

Supplementary Figure 4. Investigation of distorted synthetic polycrystals. **a** Analysis on defective version of mono-species polycrystal shown in Fig. 2a. **b** Analysis on defective version of superalloy structure shown in Fig. 2h.

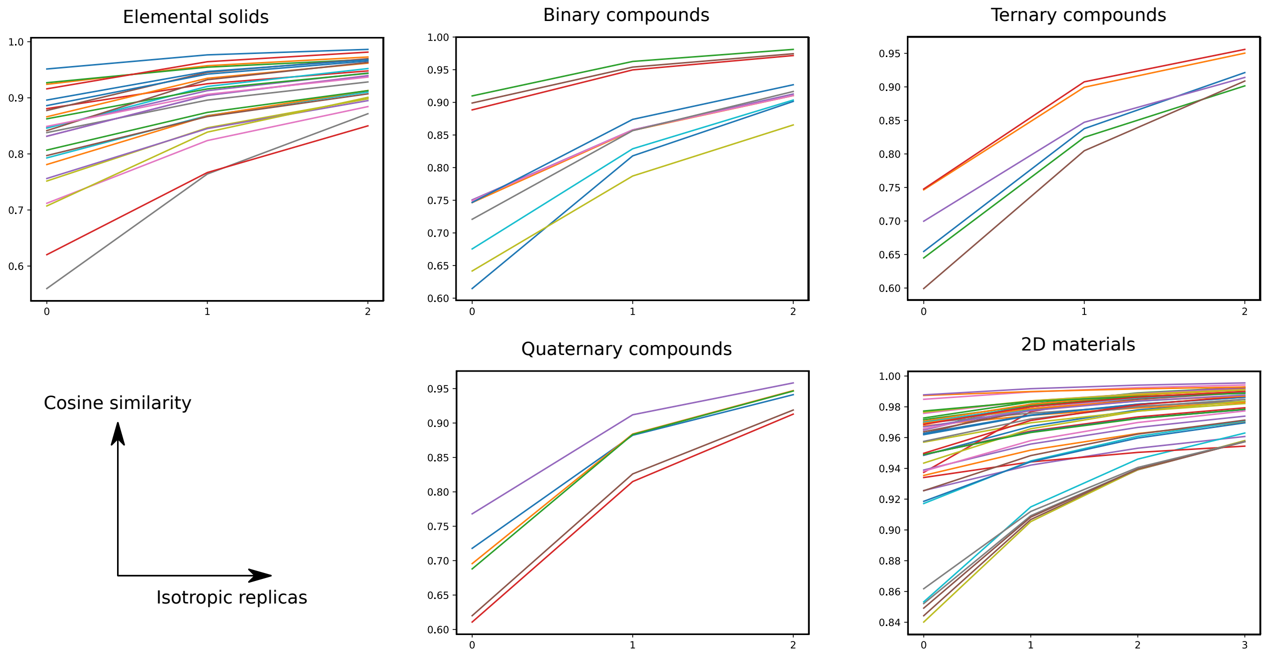

Supplementary Figure 5. Cosine similarity plots for elemental, binary, ternary, and quaternary compounds as well as 2D materials (for SOAP settings $R_C = 4.0 \cdot d_{NN}, \sigma = 0.1 \cdot d_{NN}$, and exsf $= 1.0$ corresponding to the center of the parameter range used in the training set). Each line corresponds to a particular prototype. The x-axis corresponds to three different (non-periodic) supercells, where supercell "0" stands for the smallest isotropic supercell (for instance $4 \times 4 \times 4$ repetitions) for which at least 100 atoms are obtained. Supercells "1" and "2" correspond to the next two bigger isotropic replicas (e.g., $5 \times 5 \times 5$ and $6 \times 6 \times 6$). The y-axis corresponds to the cosine similarity of the respective supercell to the periodic structure, i.e., the case of infinite replicas. One can see a continuous increase of similarity with larger supercell size, where for the largest supercell, the similarity is greater than 0.8 for all prototypes. Thus, it is to be expected that systems sizes larger than those included in the training set can be correctly classified by ARISE. For smaller systems, however, generalization ability will depend on the prototype. Practically, one can include smaller supercells in the training set, which is not a major problem due to fast convergence time.

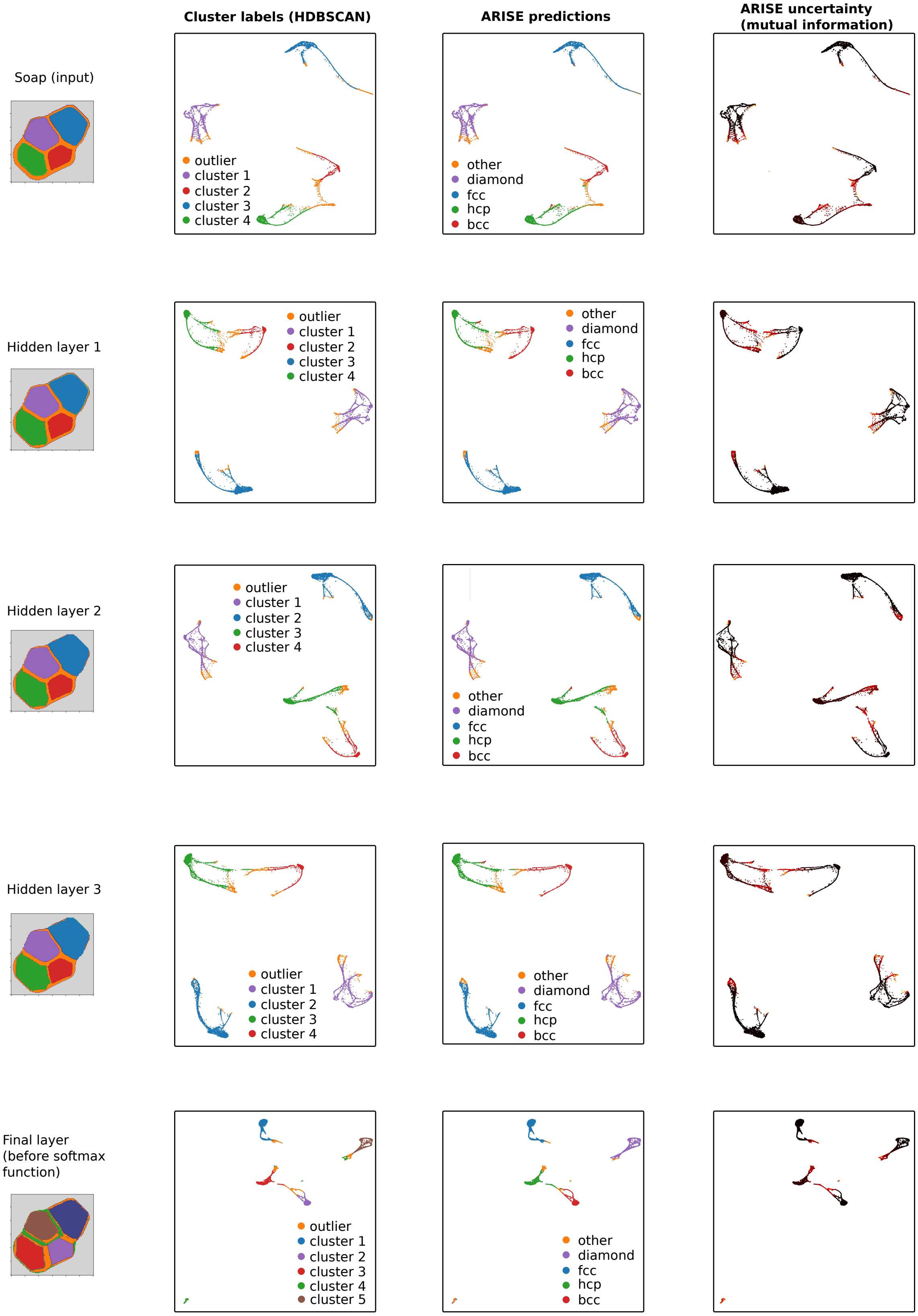

Supplementary Figure 6. Unsupervised analysis analogous to Figure 2d-g, for all layers (before the ReLU or rather the softmax function) with a minimum distance of 0.1 and a number of neighbors of 500.

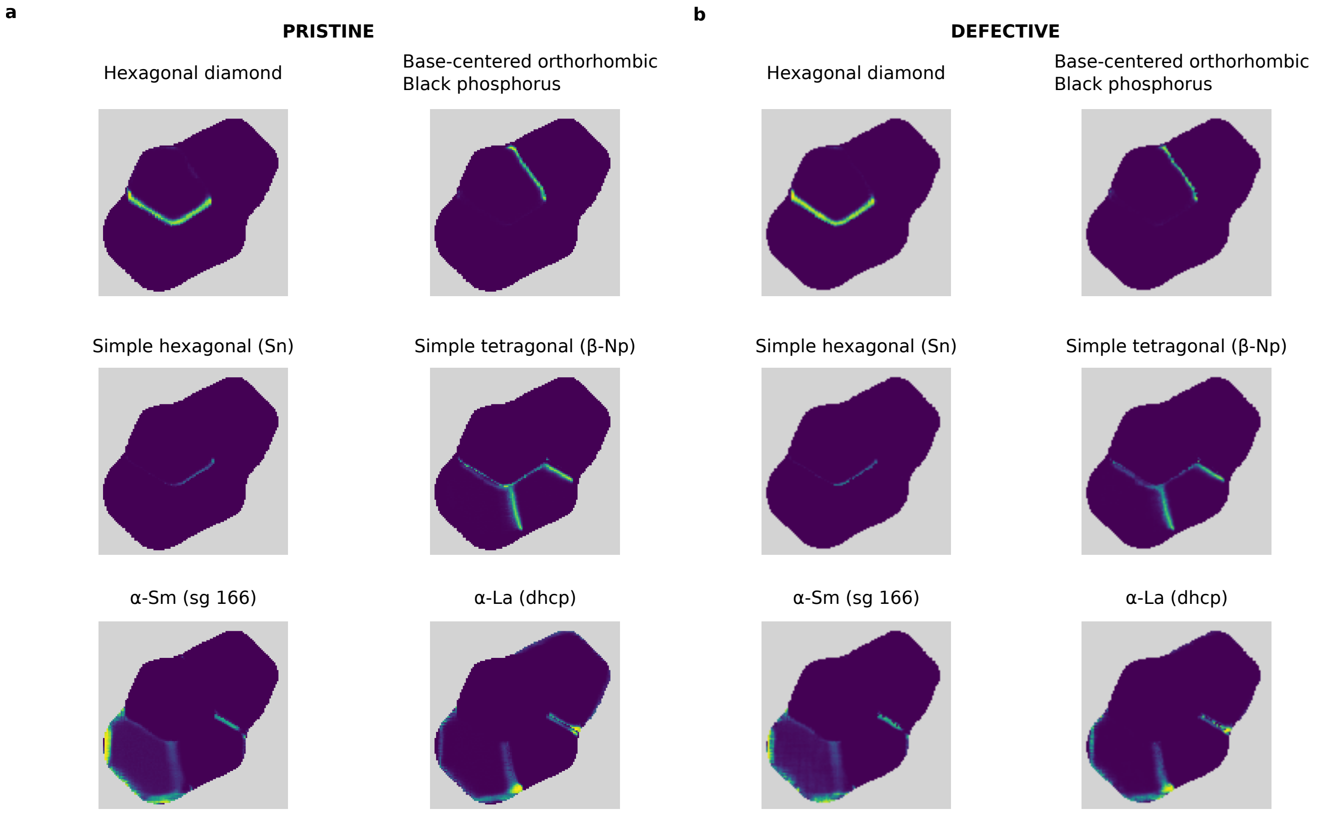

Supplementary Figure 7. Probability maps of the most important prototypes for both pristine (**a**) and defective (**b**) version of the mono-species polycrystal in Fig. 2a.

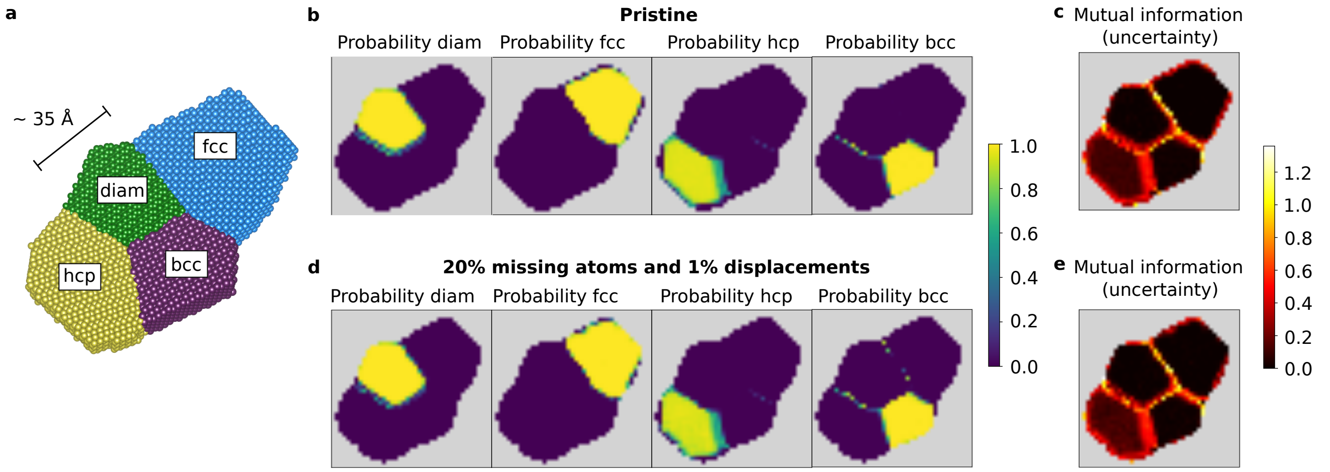

Supplementary Figure 8. Mono-species elemental polycrystal investigation via strided pattern matching using lower resolution (stride of 3.0 Åin both x and y direction opposed to 1.0 Å as in Fig. 2). Choosing the stride is a trade-off between computation time and resolution. For instance, at the grain boundary between diamond and hcp, the transition from diamond to hexagonal diamond to hcp (cf. Supplementary Fig. 7) are recognized in Fig. 2b, while being obscured in the presented low resolution pictures.

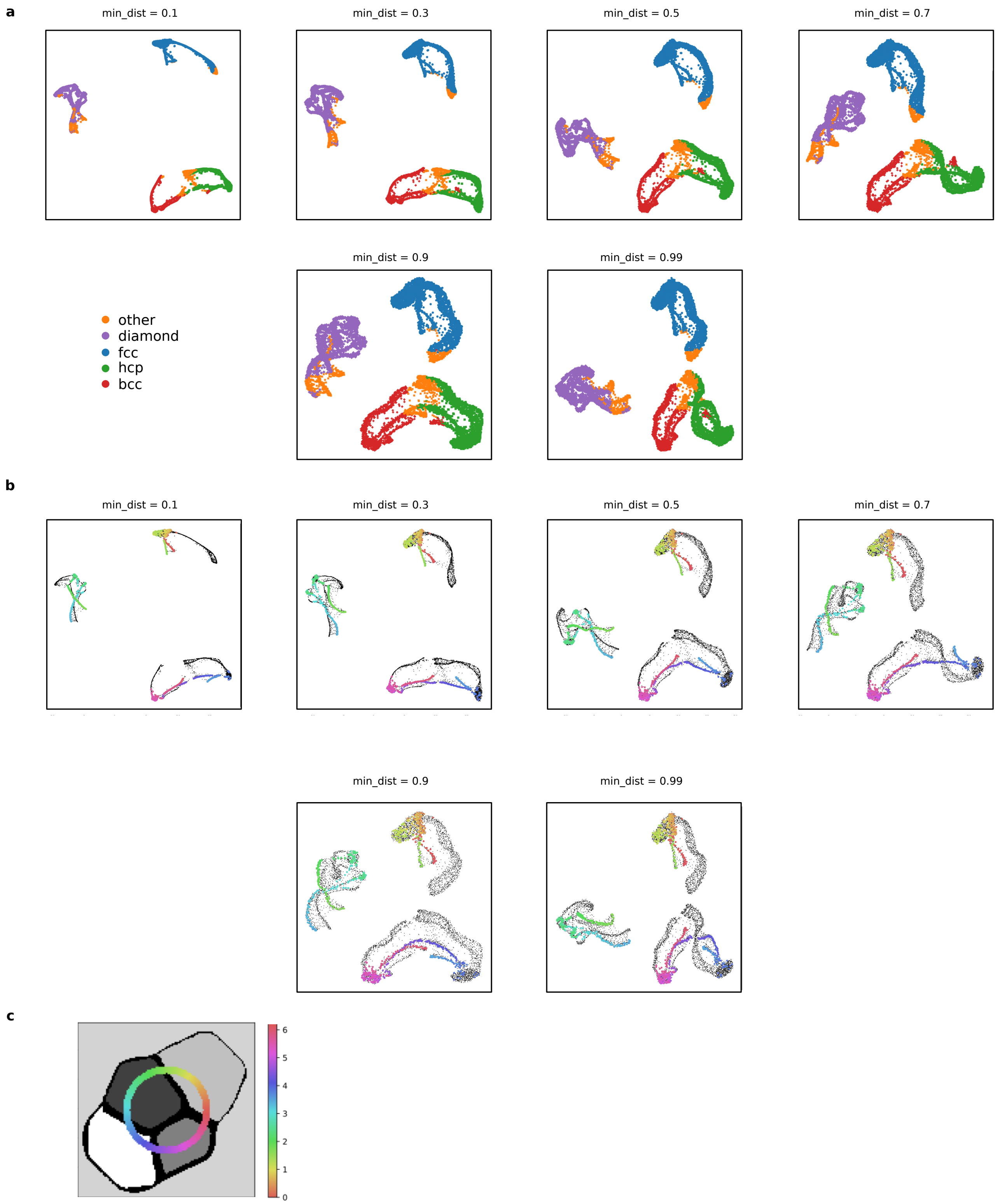

Supplementary Figure 9. Connection between UMAP embedding and real space. This figure confirms the observation that ARISE's representations of different spatial regions (crystalline regions but in particular grain boundaries, here: transitions between fcc, bcc, hcp, and diamond) are mapped to different regions in the UMAP projection. **a** Influence of the min_dist parameter in the UMAP projection (number of neighbors fixed to 500). In line with intuition, for larger min_dist, points appear more spread. In particular, connected subregions appear in the clusters, whose connection to real space is investigated in **b**: The connected strings of points actually correspond to transitions within and between crystalline regions. This is demonstrated by traversing a circle around the center of the real space structure (**c**) and coloring the embedded points according to the angle.

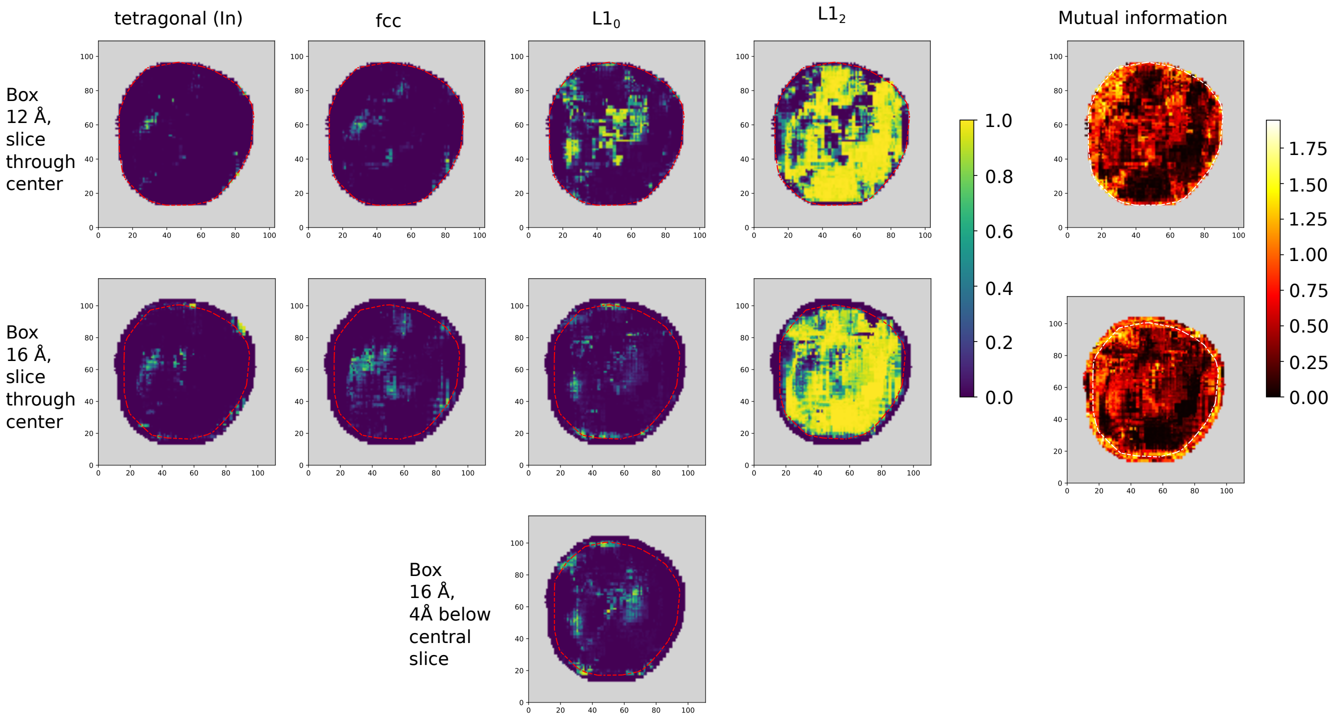

Supplementary Figure 10. Comparison of crystal maps (slice through center, most important prototypes and mutual information) for AET nanoparticle data[23] for two different box sizes. Dashed lines indicate the crystal boundaries in all 2D maps. ARISE allows to detect the appearance of the tetragonally distorted fcc prototype (In). For larger box sizes, the fcc assignment increases in the center and also the $L1_2$ classification probability rises. While the central slice of the $L1_0$ prototype for a box size of 16 Å shows only weak signal, a slice slightly below reveals higher probability (see bottom, isolated slice), i.e., ARISE does not overlook this physically relevant phase.

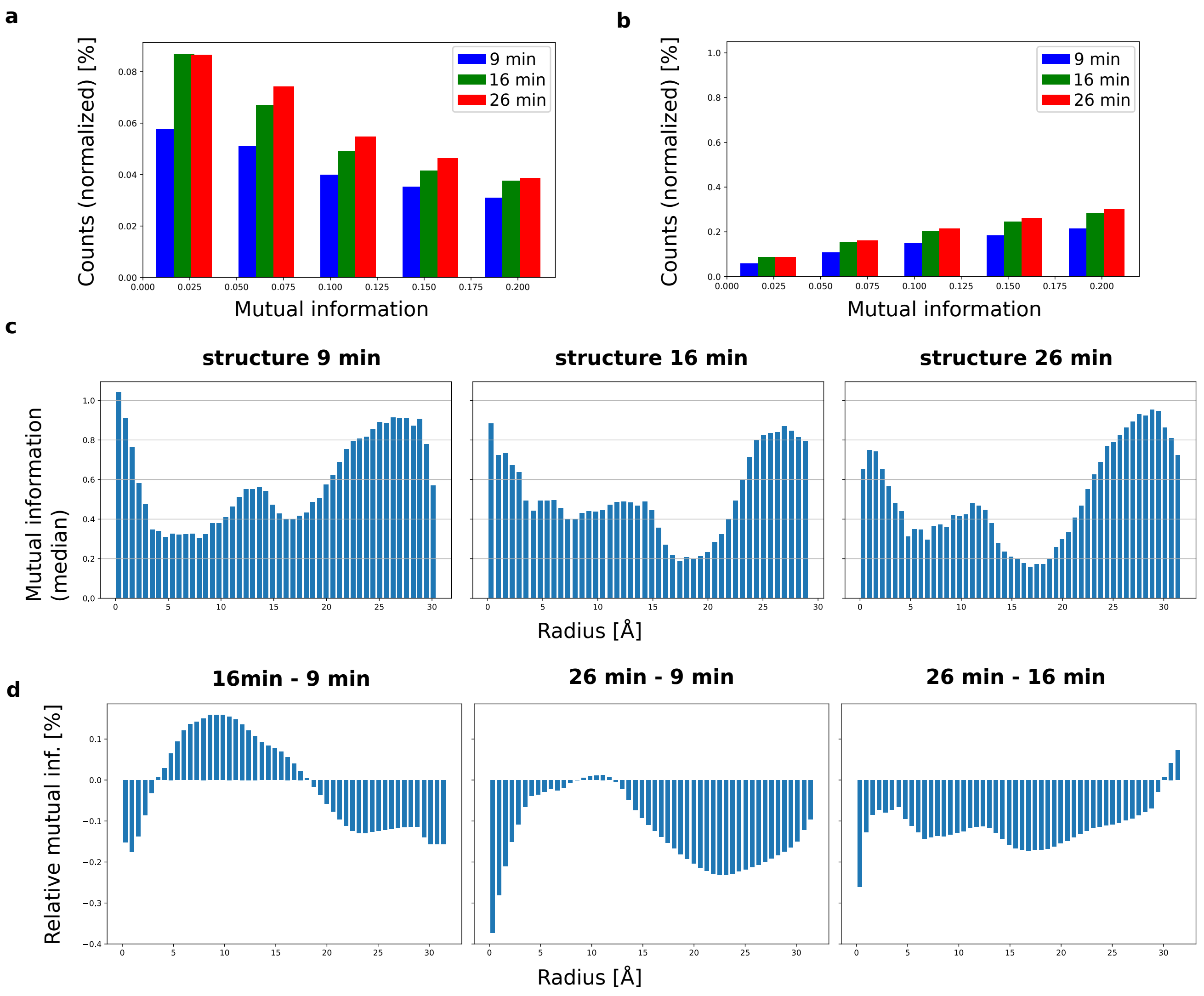

Supplementary Figure 11. Quantitative study of mutual information distribution for different annealing times. **a** Histogram of mutual information values for each annealing time (where the corresponding histograms are normalized via dividing each bin by the total number of boxes). Only mutual-information values smaller than 0.2 are shown, which correspond to the "dark", i.e., low mutual information spots in Fig. 4c. **b** Cumulative distribution calculated for the histogram shown in **a**. From **a**, **b** it is apparent that the number of low-uncertainty boxes increases for larger annealing times. **c-d** Investigation of the radial distribution of the mutual information. **c** Histograms of uncertainty (mutual information) obtained via spatially binning the SPM maps of 4**c** into spherical shells, where the median is computed for each bin. Given a mutual information value, the associated radius is calculated as the distance of the center of the corresponding box (as obtained via SPM) to the center of the most central box. **d** Each panel shows the difference between the cumulative distributions of two annealing times, where the cumulative distributions are calculated from the histograms shown in **b**. In addition the histograms are normalized the following way: Given the times t_1, t_2 with $t_1 < t_2$, the cumulative sum of $t_2 - t_1$ is calculated and then divided by the cumulative sum of time t_1 such that the fractional change from t_1 to t_2 is obtained. One can conclude that in **c** a clear decrease of mutual information can be spotted in specific regions, e.g., for the radial region 15-20 Å. The cumulative sums that are used in **d** allow to quantify the order more globally in the sense that each bin (of the cumulative sum corresponding to a specific annealing time) is proportional to the spherically averaged integral from radius zero up to the radius corresponding to the bin. Since the particle sizes are changing over time due to diffusion, the particles have different size. Thus, we single out a radius at which to compare the global order: for instance comparing the bins corresponding to a radius of r=25 Å, we see that for all three panels, the values are negative and thus the structure that has been annealed longer shows larger global order.

#	Prototype	Symmetry	Material type	Data source
1.	bcc (W)	229, cubic	Bulk, Elemental solid	AFLOW / NOMAD
2.	diamond (C)	227, cubic	Bulk, Elemental solid	AFLOW / NOMAD
3.	fcc (Cu)	225, cubic	Bulk, Elemental solid	AFLOW / NOMAD
4.	α-Po	221, (simple) cubic	Bulk, Elemental solid	AFLOW / NOMAD
5.	hcp (Mn)	194, hexagonal	Bulk, Elemental solid	AFLOW / NOMAD
6.	α-La (dhcp)	194, hexagonal	Bulk, Elemental solid	AFLOW / NOMAD
7.	Hexagonal diamond	194, hexagonal	Bulk, Elemental solid	AFLOW / NOMAD
8.	Hexagonal graphite	194, hexagonal	Bulk, Elemental solid	AFLOW / NOMAD
9.	Sn	191, (simple) hexagonal	Bulk, Elemental solid	AFLOW / NOMAD
10.	Buckled graphite	186, hexagonal	Bulk, Elemental solid	AFLOW / NOMAD
11.	α-As	166, rhombohedral	Bulk, Elemental solid	AFLOW / NOMAD
12.	α-Hg	166, rhombohedral	Bulk, Elemental solid	AFLOW / NOMAD
13.	α-Sm	166, rhombohedral	Bulk, Elemental solid	AFLOW / NOMAD
14.	β-O	166, rhombohedral	Bulk, Elemental solid	AFLOW / NOMAD
15.	β-Po	166, rhombohedral	Bulk, Elemental solid	AFLOW / NOMAD
16.	γ-Se	152, trigonal hexagonal	Bulk, Elemental solid	AFLOW / NOMAD
17.	Rhombohedral graphite	166, rhombohedral	Bulk, Elemental solid	AFLOW / NOMAD
18.	α-Pa	139, (body-centered) tetragonal	Bulk, Elemental solid	AFLOW / NOMAD
19.	β-Sn	141, (body-centered) tetragonal	Bulk, Elemental solid	AFLOW / NOMAD
20.	In	139, (body-centered) tetragonal	Bulk, Elemental solid	AFLOW / NOMAD
21.	γ-N	136, (simple) tetragonal	Bulk, Elemental solid	AFLOW / NOMAD
22.	β-Np	129, (simple) tetragonal	Bulk, Elemental solid	AFLOW / NOMAD
23.	γ-Pu	70, (face-centered) orthorhombic	Bulk, Elemental solid	AFLOW / NOMAD
24.	α-Ga	64, (base-centered) orthorhombic	Bulk, Elemental solid	AFLOW / NOMAD
25.	Black phosphorus	64, (base-centered) orthorhombic	Bulk, Elemental solid	AFLOW / NOMAD
26.	Molecular iodine	64, (base-centered) orthorhombic	Bulk, Elemental solid	AFLOW / NOMAD
27.	α-U	63, (base-centered) orthorhombic	Bulk, Elemental solid	AFLOW / NOMAD

Supplementary Table 4. Complete list of prototypes (part I) included in the training set of this work. If provided by the respective resources, information on space group, crystal system or Bravais lattice is listed.

#	Prototype	Symmetry	Material type	Data source
28.	NaCl	225, cubic	Bulk, Binary compound	AFLOW / NOMAD
29.	CsCl	221, cubic	Bulk, Binary compound	AFLOW / NOMAD
30.	$L1_2$ (Cu_3Au)	221 (simple) cubic	Bulk, Binary compound	AFLOW / NOMAD
31.	Zinc blende (ZnS)	216, (face-centered) cubic	Bulk, Binary compound	AFLOW / NOMAD
32.	FeSi	198 (simple) cubic	Bulk, Binary compound	AFLOW / NOMAD
33.	NiAs	194, hexagonal	Bulk, Binary compound	AFLOW / NOMAD
34.	Wurtzite (ZnS)	186, hexagonal	Bulk, Binary compound	AFLOW / NOMAD
35.	$L1_0$ (CuAu)	123, (simple) tetragonal	Bulk, Binary compound	AFLOW / NOMAD
36.	CrB	63, (base-centered) orthorhombic	Bulk, Binary compound	AFLOW / NOMAD
37.	MnP	62, (simple) orthorhombic	Bulk, Binary compound	AFLOW / NOMAD
38.	FeB	62, (simple) orthorhombic	Bulk, Binary compound	AFLOW / NOMAD
39.	$AgNbO_3$	cubic	Bulk, Ternary compound	CMR
40.	$CsSnI_3$	cubic	Bulk, Ternary compound	CMR
41.	$CsSnCl_3$	tetragonal	Bulk, Ternary compound	CMR
42.	Cs_2WO_4	tetragonal	Bulk, Ternary compound	CMR
43.	$Ca_3Ge_2O_7$	tetragonal	Bulk, Ternary compound	CMR
44.	$CsSnCl_3$	orthorhombic	Bulk, Ternary compound	CMR
45.	$Cu_2BaGeSe_4$	144 (trigonal)	Bulk, Quaternary compound	CMR
46.	Cu_2CdSnS_4	121 (tetragonal)	Bulk, Quaternary compound	CMR
47.	Cu_2ZnSnS_4	82 (tetragonal)	Bulk, Quaternary compound	CMR
48.	Cu_2KVS_4	40 (orthorhombic)	Bulk, Quaternary compound	CMR
49.	Cu_2CdGeS_4	31 (orthorhombic)	Bulk, Quaternary compound	CMR
50.	Cu_2ZnSiS_4	7 (monoclinic)	Bulk, Quaternary compound	CMR

Supplementary Table 5. Complete list of prototypes (part II) included in the training set of this work.

#	Prototype	Symmetry	Material type	Data source
51.	Graphene	191 (hexagonal)	2D Materials	CMR
52.	Ti_3C_2	187 (hexagonal)	2D Materials	CMR
53.	$Ti_3C_2O_2$	187 (hexagonal)	2D Materials	CMR
54.	MoS_2	187 (hexagonal)	2D Materials	CMR
55.	$Ti_3C_2H_2O_2$	187 (hexagonal)	2D Materials	CMR
56.	GaS	187 (hexagonal)	2D Materials	CMR
57.	BN	187 (hexagonal)	2D Materials	CMR
58.	$Ti_2CH_2O_2$	164 (trigonal)	2D Materials	CMR
59.	Ti_2CO_2	164 (trigonal)	2D Materials	CMR
60.	CdI_2	164 (trigonal)	2D Materials	CMR
61.	CH	164 (trigonal)	2D Materials	CMR
62.	CH_2Si	156 (trigonal)	2D Materials	CMR
63.	Ti_4C_3	156 (trigonal)	2D Materials	CMR
64.	BiTeI	156 (trigonal)	2D Materials	CMR
65.	$Ti_4C_3O_2$	156 (trigonal)	2D Materials	CMR
66.	GeSe	156 (trigonal)	2D Materials	CMR
67.	MoSSe	156 (trigonal)	2D Materials	CMR
68.	$Ti_4C_3H_2O_2$	156 (trigonal)	2D Materials	CMR
69.	$AgBr_3$	150 (trigonal)	2D Materials	CMR

Supplementary Table 6. Complete list of prototypes (part III) included in the training set of this work.

#	Prototype	Symmetry	Material type	Data source
70.	$TiCl_3$	150 (trigonal)	2D Materials	CMR
71.	BiI_3	147 (trigonal)	2D Materials	CMR
72.	FeSe	129 (tetragonal)	2D Materials	CMR
73.	PbSe	123 (tetragonal)	2D Materials	CMR
74.	GeS_2	115 (tetragonal)	2D Materials	CMR
75.	C_3N	65 (orthorhombic)	2D Materials	CMR
76.	FeOCl	59 (orthorhombic)	2D Materials	CMR
77.	P	28 (orthorhombic)	2D Materials	CMR
78.	PdS_2	14 (monoclinic)	2D Materials	CMR
79.	MnS_2	14 (monoclinic)	2D Materials	CMR
80.	GaSe	12 (monoclinic)	2D Materials	CMR
81.	TiS_3	11 (monoclinic)	2D Materials	CMR
82.	WTe_2	11 (monoclinic)	2D Materials	CMR
83.	HfBrS	7 (monoclinic)	2D Materials	CMR
84.	RhO	6 (monoclinic)	2D Materials	CMR
85.	SnS	6 (monoclinic)	2D Materials	CMR
86.	NiSe	6 (monoclinic)	2D Materials	CMR
87.	AuSe	6 (monoclinic)	2D Materials	CMR

Supplementary Table 7. Complete list of prototypes (part IV) included in the training set of this work.

#	Prototype	Symmetry	Material type	Data source
88.	VTe_3	6 (monoclinic)	2D Materials	CMR
89.	ReS_2	2 (monoclinic)	2D Materials	CMR
90.	$ScPSe_3$	1 (triclinic)	2D Materials	CMR
91.	PbA_2I_4	1 (triclinic)	2D Materials	CMR
92.	PbS	1 (triclinic)	2D Materials	CMR
93.	CrW_3S_8	1 (triclinic)	2D Materials	CMR
94.	$VPSe_3$	1 (triclinic)	2D Materials	CMR
95.	$CrWS_4$	1 (triclinic)	2D Materials	CMR
96.	$MnPSe_3$	1 (triclinic)	2D Materials	CMR
97.	Carbon nanotube	armchair, (3,3), 30.0°, 4.07 Å	Nanotubes, mono-species	ASE
98.	Carbon nanotube	armchair, (4,4), 30.0°, 5.42 Å	Nanotubes, mono-species	ASE
99.	Carbon nanotube	chiral, (4,2), 19.11°, 4.14 Å	Nanotubes, mono-species	ASE
100.	Carbon nanotube	chiral, (4,3), 25.28°, 4.76 Å	Nanotubes, mono-species	ASE
101.	Carbon nanotube	chiral, (5,1), 8.95°, 4.36 Å	Nanotubes, mono-species	ASE
102.	Carbon nanotube	chiral, (5,2), 16.1°, 4.89 Å	Nanotubes, mono-species	ASE
103.	Carbon nanotube	chiral, (5,3), 21.79°, 5.48 Å	Nanotubes, mono-species	ASE
104.	Carbon nanotube	chiral, (6,1), 7.59°, 5.13 Å	Nanotubes, mono-species	ASE
105.	Carbon nanotube	chiral, (6,2), 13.9°, 5.65 Å	Nanotubes, mono-species	ASE
106.	Carbon nanotube	chiral, (7,1), 6.59°, 5.91 Å	Nanotubes, mono-species	ASE
107.	Carbon nanotube	zigzag, (6,0), 0.0°, 4.7 Å	Nanotubes, mono-species	ASE
108.	Carbon nanotube	zigzag, (7,0), 0.0°, 5.48 Å	Nanotubes, mono-species	ASE

Supplementary Table 8. Complete list of prototypes (part V) included in the training set of this work. For the carbon nanotubes, the symmetry column specifies the configuration type (chiral, zigzag or armchair) together with the corresponding chiral numbers (n,m), the chiral angle θ and the nanotube diameter.

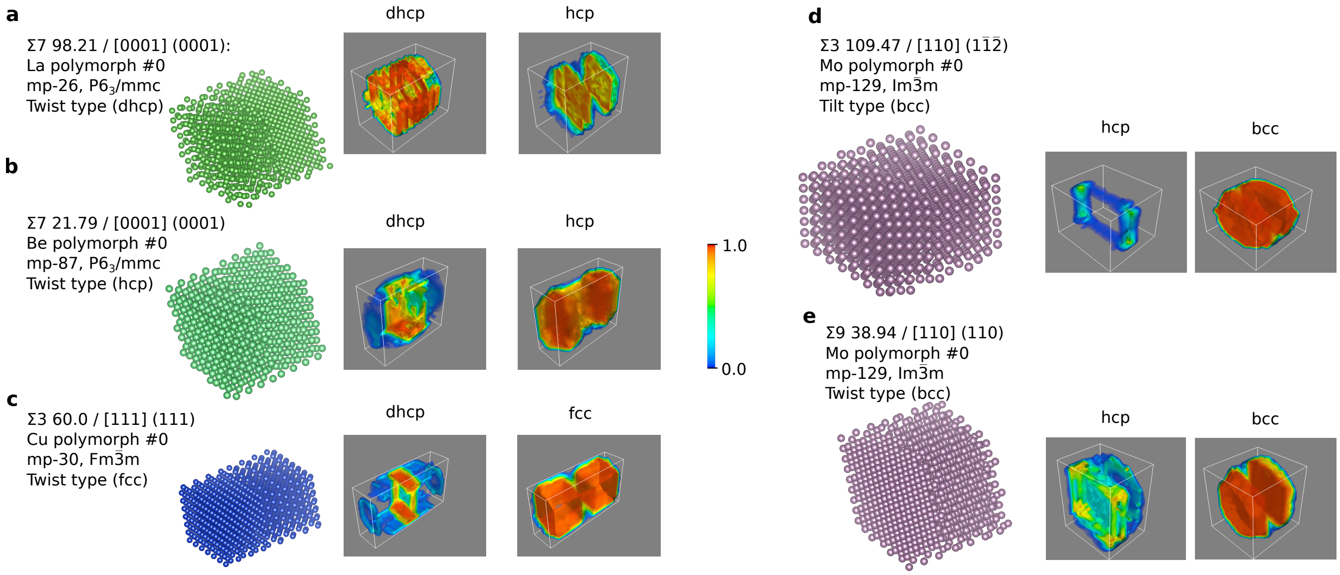

Supplementary Figure 12. Five representative examples from the grain boundary database (GBDB), which is the largest, currently available database of DFT-computed grain boundary properties[24]. This database can be accessed via Materials Project or `http://crystalium.materialsvirtuallab.org/`. For each structure, four lines of information are provided: The first line specifies the information that is required to uniquely describe a grain boundary structure[25], where first the Σ-parameter is given, followed by rotation angle, rotation axis and grain-boundary plane. The relative orientation of two neighboring grains is described by three degrees of freedom (rotation angle and axis). The two degrees of freedom specified via the grain-boundary plane complete the unique characterization of a grain-boundary structure. The second line specifies the element and the entry number of the polymorph in the database (for a given element, multiple grain boundaries can be available). The third line specifies the materials project ID and the space group. The last line specifies the grain-boundary type (twist, tilt) alongside the dominating crystal structure. The database entries correspond to periodic cells that contain a grain boundary. We replicate this initial cell isotropically (in the plane parallel to the grain boundary) until at least 1000 atoms are contained in the structure. For all examples, the dominating phase and grain boundary regions are correctly detected as shown via the 3D classification probability maps of the most popular assignments according to ARISE. These selected structures illustrate the advantages of ARISE in the following way: **a** shows that ARISE can detect dhcp symmetry in a polycrystal. In particular, the close-packing corresponding to dhcp cannot be classified in comparable automatic fashion by any of the available methods. For hcp (**b**) and fcc (**c**), the dhcp assignments only appear at the grain boundary. **d** and **e** are two different grain boundary types that do not only differ in their defining degrees of freedom but also are of tilt (**d**) and twist (**e**) type. ARISE distinguishes the local structures at the grain boundary which is indicated by its assignments: while for the twist type (**e**) hcp is the dominating assignment at the grain boundary, for the tilt type the hcp probability drops to zero at the grain boundary (except for the outer borders). The following SPM parameters are chosen for all examples: A stride of 2 Å suffices to resolve the main characteristics. For a box size of 16 Å at least 100 atoms are contained in the boxes within the grains.

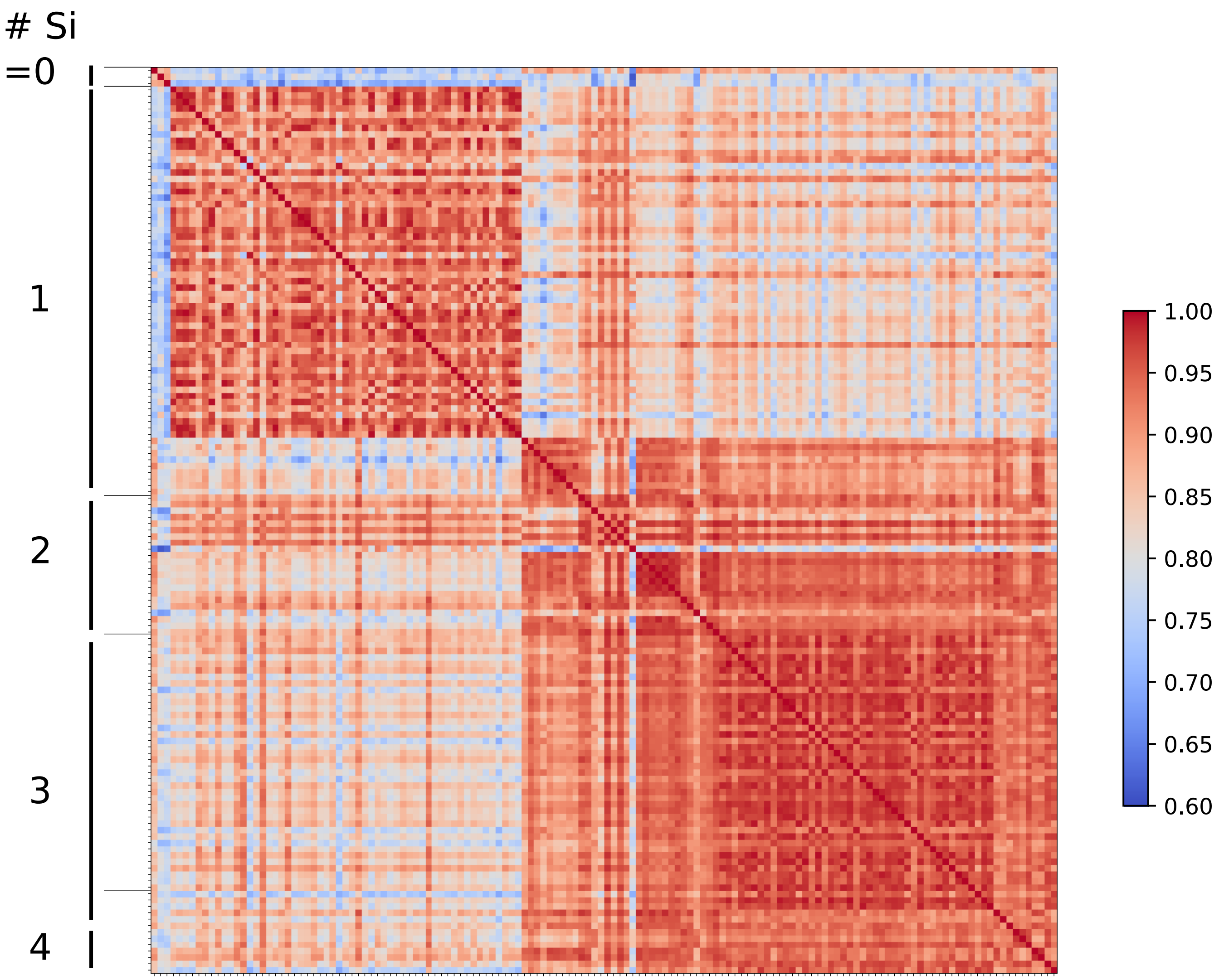

Supplementary Figure 13. Cross similarity matrix for a selection of the defect library[26] that is larger than in the main text (Fig. 3d). Specifically, 140 structures as well as the mono-species structures from Fig. 3a (right),e are considered. For reconstruction of atomic positions, Atomnet is employed, where for the structures from the library, atomic positions are reconstructed using a model that can also classify the chemical species. We employed the model that is available at https://github.com/pycroscopy/AICrystallographer/tree/master/DefectNet.

Supplementary references

1. Bartók, A. P., Kondor, R. & Csányi, G. On representing chemical environments. *Phys. Rev. B* **87**, 184115 (2013).
2. Mavračić, J., Mocanu, F. C., Deringer, V. L., Csányi, G. & Elliott, S. R. Similarity between amorphous and crystalline phases: The case of TiO2. *The J. Phys. Chem. Lett.* **9**, 2985–2990 (2018).
3. De, S., Bartók, A. P., Csányi, G. & Ceriotti, M. Comparing molecules and solids across structural and alchemical space. *Phys. Chem. Chem. Phys.* **18**, 13754–13769 (2016).
4. Gal, Y. & Ghahramani, Z. Dropout as a Bayesian approximation: Representing model uncertainty in deep learning. In *International Conference on Machine Learning*, 1050–1059 (2016).
5. Hinton, G. E., Srivastava, N., Krizhevsky, A., Sutskever, I. & Salakhutdinov, R. R. Improving neural networks by preventing co-adaptation of feature detectors. *arXiv preprint arXiv:1207.0580* (2012).
6. Srivastava, N., Hinton, G., Krizhevsky, A., Sutskever, I. & Salakhutdinov, R. Dropout: a simple way to prevent neural networks from overfitting. *The J. Mach. Learn. Res.* **15**, 1929–1958 (2014).
7. Houlsby, N., Huszár, F., Ghahramani, Z. & Lengyel, M. Bayesian active learning for classification and preference learning. *arXiv preprint arXiv:1112.5745* (2011).
8. Bergstra, J. S., Bardenet, R., Bengio, Y. & Kégl, B. Algorithms for hyper-parameter optimization. In *Advances in Neural Information Processing Systems*, 2546–2554 (2011).
9. Bergstra, J., Yamins, D. & Cox, D. D. Making a Science of Model Search: Hyperparameter Optimization in Hundreds of Dimensions for Vision Architectures. In *Proceedings of the 30th International Conference on International Conference on Machine Learning - Volume 28*, ICML'13, I–115–I–123 (JMLR.org, 2013).
10. Pandey, M. & Jacobsen, K. W. Promising quaternary chalcogenides as high-band-gap semiconductors for tandem photoelectrochemical water splitting devices: A computational screening approach. *Phys. Rev. Mater.* **2**, 105402 (2018).
11. Stukowski, A. Visualization and analysis of atomistic simulation data with OVITO–the Open Visualization Tool. *Model. Simul. Mater. Sci. Eng.* **18**, 015012 (2009).
12. Bain, E. C. & Dunkirk, N. Y. The nature of martensite. *trans. AIME* **70**, 25–47 (1924).
13. Zhao, J.-C. & Notis, M. R. Continuous cooling transformation kinetics versus isothermal transformation kinetics of steels: a phenomenological rationalization of experimental observations. *Mater. Sci. Eng. R: Reports* **15**, 135–207 (1995).
14. Grimvall, G., Magyari-Köpe, B., Ozoliņš, V. & Persson, K. A. Lattice instabilities in metallic elements. *Rev. Mod. Phys.* **84**, 945 (2012).
15. Alippi, P., Marcus, P. M. & Scheffler, M. Strained tetragonal states and bain paths in metals. *Phys. Rev. Lett.* **78**, 3892–3895 (1997). DOI 10.1103/PhysRevLett.78.3892.
16. Buschbeck, J. *et al.* Full Tunability of Strain along the fcc-bcc Bain Path in Epitaxial Films and Consequences for Magnetic Properties. *Phys. Rev. Lett.* **103**, 216101 (2009). DOI 10.1103/PhysRevLett.103.216101.
17. Haftel, M. I. & Gall, K. Density functional theory investigation of surface-stress-induced phase transformations in fcc metal nanowires. *Phys. Rev. B - Condens. Matter Mater. Phys.* **74**, 035420 (2006). DOI 10.1103/PhysRevB.74.035420.
18. Davey, W. P. The lattice parameter and density of pure tungsten. *Phys. Rev.* **26**, 736 (1925).
19. Straumanis, M. & Yu, L. Lattice parameters, densities, expansion coefficients and perfection of structure of Cu and of Cu–In α phase. *Acta Crystallogr. Sect. A: Cryst. Physics, Diffraction, Theor. Gen. Crystallogr.* **25**, 676–682 (1969).
20. Deshpande, V. & Pawar, R. Anisotropic thermal expansion of indium. *Acta Crystallogr. Sect. A: Cryst. Physics, Diffraction, Theor. Gen. Crystallogr.* **25**, 415–416 (1969).
21. Zachariasen, W. On the crystal structure of protactinium metal. *Acta Crystallogr.* **12**, 698–700 (1959).
22. Isayev, O. *et al.* Materials cartography: representing and mining materials space using structural and electronic fingerprints. *Chem. Mater.* **27**, 735–743 (2015).
23. Yang, Y. *et al.* Deciphering chemical order/disorder and material properties at the single-atom level. *Nat.* **542**, 75–79 (2017).
24. Zheng, H. *et al.* Grain boundary properties of elemental metals. *Acta Materialia* **186**, 40–49 (2020).
25. Lejček, P. Grain boundaries: description, structure and thermodynamics. In *Grain boundary segregation in metals*, 5–24 (Springer, 2010).
26. Ziatdinov, M. *et al.* Building and exploring libraries of atomic defects in graphene: Scanning transmission electron and scanning tunneling microscopy study. *Sci. advances* **5**, eaaw8989 (2019).
